# Endpoints and viewpoints on spatial neglect

**DOI:** 10.1111/jnp.12278

**Published:** 2022-05-04

**Authors:** Robert D. McIntosh, Sumio Ishiai

**Affiliations:** ^1^ Human Cognitive Neuroscience, Psychology University of Edinburgh Edinburgh UK; ^2^ Department of Rehabilitation Medicine Sapporo Medical University School of Medicine Sapporo Japan

**Keywords:** attention, bisection, endpoint weightings, mental representation, neglect

## Abstract

In this issue of the *Journal of Neuropsychology*, Abe and Ishiai (2022) report an experiment designed to probe the subjective experience of line bisection in neglect. A re‐analysis of their data can also offer insights into how best to characterise neglect performance for this and other tasks. We show that sensitive measures of neglect can be obtained by quantifying the difference in the influence (or ‘weighting’) that each endpoint has on the response. The right endpoint is dramatically more influential than the left in people with neglect performing line bisection and endpoint reproduction tasks. This supports the view that neglect may limit the ability to simultaneously represent two locations, so that the response is determined primarily with respect to the right endpoint. We also discuss Abe and Ishiai's conclusion that bisection responses in neglect are accompanied by the subjective experience of a complete line extending equally to either side of the chosen midpoint.

## BACKGROUND

If a person with left spatial neglect, following stroke to the right side of the brain, is asked to bisect a horizontal line, they will typically deviate to the right of the true midpoint. The line bisection task has long been used to diagnose and study neglect, yet it is imperfectly understood. There is some debate about how best to measure performance on this apparently simple task, and there is an enduring mystery around what the person with neglect experiences when they make a bisection response. For more than thirty years, Ishiai and colleagues have argued that bisection responses in left neglect are based on limited information from a rightward portion of the line, but are accompanied by the experience of a complete line extending to both sides of the subjective midpoint (Ishiai et al., 1989, 1992, 1995, 2006).

In this issue of the *Journal of Neuropsychology*, Abe and Ishiai (2022) report an experiment designed to probe the subjective experience of bisection in neglect. They used a novel ‘endpoint reproduction’ task, performed with a stylus on a touch panel. First, the participant is shown a horizontal line (25, 100 or 250 mm in length) and asked to mark its midpoint, which is the standard bisection task. The line then disappears, and the participant is asked to touch the remembered position of the left or the right endpoint. The left endpoints reproduced by people with neglect were unrelated to the real left endpoint, but were well‐predicted by the distance between the bisection response and the right endpoint. This is consistent with the idea that when a person with left neglect (mis‐)bisects a horizontal line, they are ignorant of its true leftward extent, but subjectively see it extending equally to either side of their chosen midpoint.

Although it was not their focus, Abe and Ishiai's (2022) participant‐level data (as reported in their supplementary tables) also offer insights into how best to measure neglect performance, for line bisection and for the endpoint reproduction task. These insights emerged during the review process but did not fit neatly into the published paper, so we have collaborated on this commentary to summarise them. The analysis to be presented shows that sensitive measures of neglect can be obtained by quantifying the difference in the influence (or ‘weighting’) that each endpoint has on the response. The right endpoint is dramatically more influential than the left in people with neglect performing line bisection and endpoint reproduction tasks. This supports the view, which we hold in common (Koyama et al., 1997; McIntosh, 2006; McIntosh et al., 2005), that people with neglect may be unable to simultaneously represent both (real) endpoints, and so their response is determined primarily or exclusively with respect to the right endpoint.

## ENDPOINT WEIGHTINGS MODEL OF LINE BISECTION

Ishiai's group were the first to notice that the bisection responses of people with severe neglect tended to maintain a relatively invariant distance from the right endpoint of the line, regardless of the line's length or position relative to the body (Koyama et al., 1997). They suggested that bisection responses, at least in severe cases of neglect, are determined the right endpoint alone. McIntosh et al. (2005) further developed this idea and tested it by devising a new format for the line bisection task. In this new format, the left and the right endpoint positions were varied independently across trials, and the focus was not on the deviation of the response from the objective midpoint, but on the degree to which the response position was predicted by the left and right endpoint positions separately. To quantify these influences, left and right ‘endpoint weightings’ were extracted, which are equal to the coefficients of the best‐fitting straight line relating the response position (P) to the left and right endpoint positions (L and R), all coded as horizontal coordinates in an environmental reference frame.^1^

McIntosh et al. (2005) showed that the weighting for the left endpoint was close to zero for people with severe neglect, meaning that it has little or no influence on behaviour. This was the extreme end of a *general* pattern that, across the spectrum of neglect severities, the weighting for the left endpoint was always lower than that for the right. The asymmetry can be captured by a simple composite measure, endpoint weightings bias (EWB), which is the right endpoint weighting minus the left endpoint weighting. EWB is positive in left neglect, indicating a dominant influence of the right endpoint. EWB is more sensitive to neglect than is the traditional measure of directional bisection error (DBE) and is more strongly related to neglect on other diagnostic tasks such as target cancellation or figure copying (McIntosh et al., 2005, 2017). It has thus been proposed as a better measure of bisection bias (McIntosh et al., 2005; Mitchell *et al*., 2022) and possibly as one of the best measures of the core bias of spatial neglect (McIntosh et al., 2017).

## ENDPOINT WEIGHTINGS IN A BISECTION TASK

**FIGURE 1:**
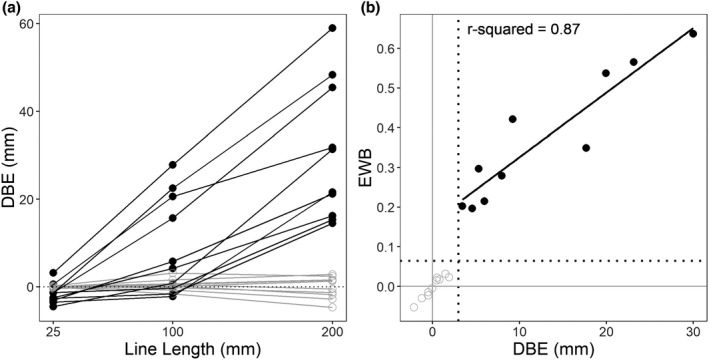
(a) Relationship between line length and mean bisection error for neglect (black) and control (grey) participants. (b) Relationship between the traditional index of directional bisection error (DBE) and the alternative index of endpoint weighting bias (EWB), with neglect shown in black and control shown in grey. The fit line is for the neglect group only. The dotted line on each axis is the upper cut‐off for left neglect, derived from the control sample by the modified‐t method (Crawford & Howell, 1998)

For a full endpoint weightings analysis to be possible, the task must be administered with the left and right endpoint positions varied independently, but this has almost never been done. Instead, the standard manipulations are to vary the length and/or the spatial position of the lines, which involves correlated changes at both endpoints. For instance, a change in line length implies symmetrical changes in the left and right endpoints. When line length is manipulated, it is found that the rightward bisection errors of neglect are largest for long lines (e.g. 200 mm), more modest for shorter lines (e.g. 100 mm), and may even ‘cross‐over’ to become leftward if very short lines are used (e.g. 25 mm; Halligan & Marshall, 1988; Marshall & Halligan, 1989). Abe and Ishiai (2022) varied line length in their study, and the expected dependence of bisection error on line length can be seen for all members of the neglect group, with a cross‐over effect for the shortest line in most cases (Figure 1a).

Abe and Ishiai's experiment did not deconfound left and right endpoint positions, so it is not possible to extract separate left and right endpoint weightings from their bisection data. But, it is still possible to estimate the endpoint weightings measure of bias, EWB, because this is always equal to two times the slope of the best‐fitting straight line relating DBE to line length within a traditional task analysis (McIntosh et al., 2005). To appreciate this, consider that a positive EWB means that bisection responses track the right endpoint more closely than the left. If the line is extended equally at both ends, and if the response tracks the right endpoint more than the left, then the net outcome will be more rightward responses for longer lines. A positive EWB, therefore, implies a positive slope between DBE and line length.^2^ So, although Abe and Ishiai (2022) did not vary the left and right endpoints independently, we can still recover EWB from the slope of the relationships plotted in Figure 1a.

Figure 1b shows mean DBE on the *x*‐axis and the recovered estimate of EWB on the *y*‐axis. These two measures of bias are closely related, which is unsurprising given that they are extracted from the same set of responses. But, the separation of neglect and control groups is much clearer for EWB (*y*‐axis) than for DBE (*x*‐axis), replicating previous demonstrations of the higher sensitivity of EWB to neglect (McIntosh et al., 2005, 2017). Saying that EWB is better at distinguishing neglect from controls in Figure 1b, is equivalent to saying that, in Figure 1a, the groups are better distinguished by the slopes of the relationship between line length and DBE than by mean levels of DBE. In fact, during the late 1980s, Halligan and Marshall did emphasise the slope of the line length effect as an excellent indicator of neglect severity (Adair et al., 1998; Halligan & Marshall, 1988; Marshall & Halligan, 1989). The endpoint weightings model provides a compelling explanation for why this is the case: because EWB (and thus the line length effect) quantifies the difference in the influence of the right and left ends of the line.

As well as being more sensitive to neglect, EWB is a proportional measure, which is inherently standardised and comparable across experiments. EWB reflects the way that the response changes across stimuli, and is independent of the particular lines used, whereas the traditional measure of DBE is heavily contingent on the particular lines. Figure 1a suggests that the sensitivity of DBE begins to approach that of EWB only for long stimulus lines (≥200 mm). In practical terms, if testing time is limited so that only a few bisection responses can be collected, then DBE may be an adequate measure of bias, provided that long enough lines are used. However, if there is an opportunity to collect multiple bisection responses, then left and right endpoints should be varied independently, so that an endpoint weightings analysis can be performed. At a theoretical level, the endpoint weightings model prompts us to rethink what is measured in line bisection, focusing on the ability to simultaneously represent two lateralised stimuli (endpoints). This may be closer to the core of neglect (and extinction) than traditional conceptions of line bisection in terms of the perception of length (McIntosh et al., 2017).

## ENDPOINT WEIGHTINGS IN AN ENDPOINT REPRODUCTION TASK

If a core component of neglect is an inability to simultaneously represent two lateralised stimuli, then Abe and Ishiai's (2022) endpoint reproduction task should also be well‐suited to measure it. The participant cannot know which endpoint the examiner will ask them to reproduce, so they must try to hold both in mind. This puts the two endpoints into a competition for resources, which the right endpoint should win easily if the person has left neglect. If Abe and Ishiai (2022) had designed their task for an endpoint weightings analysis, they would have varied the left and right endpoint locations independently, to prevent either endpoint providing clues about the location of the other. But, even without this design feature, the task is still amenable to an endpoint weightings analysis, in which the slope of the straight line relating the real and reproduced endpoint is taken to quantify the fidelity of its representation. This insight inspired the analysis of ‘Relationships between the locations of the objective and reproduced endpoints’, reported in the original paper (pp. 8–10).

**FIGURE 2:**
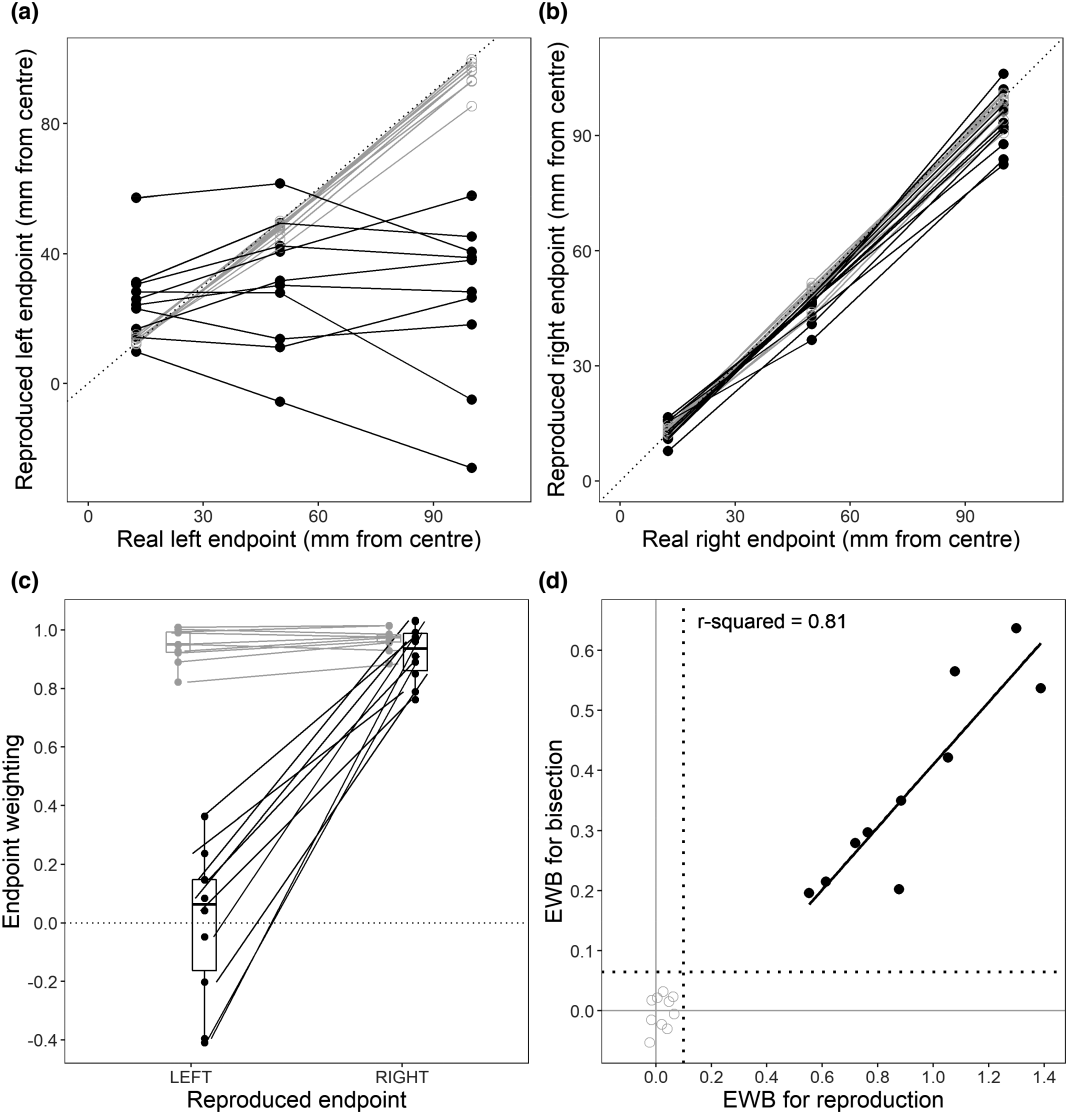
(a) and (b) are re‐plotted from figure 3 of Abe and Ishiai (2022), showing the relationship of the real and reproduced left and right endpoints for neglect (black) and control (grey) participants. (c) Endpoint weightings for the left and right endpoints, derived from reproduction responses. (d) Relationship between endpoint weightings bias (EWB) for the reproduction and bisection tasks. The fit line is for the neglect group only. The dotted line on each axis is the upper cut‐off for left neglect, derived from the control sample by the modified‐t method (Crawford & Howell, 1998). It is worth noting that the range of the *x*‐axis in panel d is approximately twice that of the *y*‐axis. This stems from the fact that the optimal endpoint weighting for reproduction responses (1) is twice that of the optimal endpoint weighting for bisection responses (0.5)

Figure 2a and b re‐plot the data from Abe and Ishiai's figure 3, showing that whilst people with neglect reproduced the right endpoint location with broadly similar fidelity to controls, there was little or no relationship between the real and reproduced left endpoints. Figure 2c re‐expresses these patterns in terms of endpoint weightings, where a weighting of one indicates perfect tracking of the endpoint position, and a weighting of zero indicates no tracking at all. The neglect group are highly sensitive to the position of the right endpoint, and wholly insensitive to the position of the left endpoint. This asymmetry can be captured as an endpoint weightings bias, given by the subtraction of the left endpoint weighting from the right; we refer to this as EWBr (for reproduction) to distinguish it from EWB for bisection.

Figure 2d shows that EWB and EWBr are tightly related (*r* ^2^ = .81). This is impressive, considering that they are derived from such different responses: EWB is extracted from bisection responses made with the stimulus line present and EWBr from endpoint reproductions after the line has disappeared. Their tight correspondence suggests that these two measures could be tapping into a common, core component of neglect. The separation between neglect and control groups is even cleaner for EWBr (*x*‐axis) than for EWB (*y*‐axis) (see also Table 1). This difference may be due to the disappearance of the stimulus line: the left endpoint has some (limited) power to influence responses whilst the line is present for bisection, but this influence may dissipate entirely once the line disappears.

**TABLE 1:** Summary measures of bias for line bisection and endpoint reproduction tasks, for neglect and control groups (*n* = 10 per group)

Task	Measure	Neglect mean (*SD*)	Control mean (*SD*)	Hedges g*_s_ [95% CIs]
Bisection	DBE	12.72 (9.26)	0.08 (1.22)	1.93 [1.23, 2.62]
	EWB	0.37 (0.16)	0.00 (0.03)	3.23 [2.28, 4.17]
Reproduction	EWBr	0.92 (0.28)	0.02 (0.03)	4.57 [3.31, 5.82]

The standardised effect size is hedges g*_s_, which gives an unbiased estimate of effect size for a comparison between two groups with unequal variances (Delacre et al., 2021). All effect sizes are extremely large, but those for the endpoint weightings measures are far higher than that those for DBE, and highest of all for the reproduction task.

DBE = directional bisection error in mm; EWB = endpoint weightings bias; EWBr = endpoint weightings bias for the endpoint reproduction task.

## CONCLUSION

Rich insights follow from viewing Abe and Ishiai's (2022) data through the lens of an endpoint weightings model. This re‐analysis has suggested that a core component of neglect is a reduced ability to represent two lateralised stimuli (endpoints), and that the endpoint reproduction task may tap into this component even better than the line bisection task does. These analyses are exploratory and based on a small sample, so further work will be needed to confirm and extend them. The endpoint reproduction task would be optimised for an endpoint weightings approach by the independent manipulation of left and right endpoint positions. It could also be adapted in various ways to broaden the scope of study. For instance, it might be interesting to compare performance when the two positions to be reproduced are endpoints of a line, or are isolated dots, to investigate whether a line is processed differently than the endpoint locations that geometrically define it.

This last point highlights a theoretical tension running through this commentary. The endpoint weightings analysis suggests that neglect involves a reduced ability to represent two lateralised stimuli (endpoints). This seems to be at odds with Abe and Ishiai's (2022) conclusion that people with neglect subjectively experience a complete line, which has two endpoints by definition. Abe and Ishiai's favoured explanation is that neglect impedes the processing of the *real* left endpoint, but a representation of a complete line is extrapolated from the attended rightward portion. The reproduced left endpoint would then be a true recollection of a perceived endpoint, but uninformed by the real left endpoint. An alternative possibility is that a person with substantial neglect is unable to sustain an overview of a complete line, whether real or imagined, because they cannot hold two endpoints in mind at once (Kinsbourne, 1993; McIntosh et al., 2005). If so, the reproduced left endpoint would not be a recollection of a prior perception, but a reconstruction from an accurate memory of the right endpoint. Whether such nuances could be captured reliably by first‐person report is unclear, and to test between these accounts may require clever experimentation, perhaps with additional functional imaging. This empirical loose end reminds us that, whilst we may improve the objective measurement of the disorders that follow brain damage, understanding the first‐person perspective is a distinct and deeper challenge.

## CONFLICT OF INTEREST

The authors declare no conflict of interest.

## AUTHOR CONTRIBUTIONS

**Robert D McIntosh:** Conceptualization; methodology; visualization; writing. **Sumio Ishiai:** Conceptualization; investigation; methodology; review and editing.

## Data Availability

Open data and analysis scripts for this article are available at https://osf.io/5mtr8/

## Footnotes

1 The centre of the workspace is usually taken to define the zero coordinate of this reference frame, though any fixed environmental point could be used. It is critical to note that the response position (P) is coded as a horizontal spatial position, not as a deviation relative to (the centre of) the stimulus line.

2 See figure 7 of McIntosh et al., 2005, for a graphical depiction of this equivalence.
